# *Alkbh5* plays indispensable roles in maintaining self-renewal of hematopoietic stem cells

**DOI:** 10.1515/med-2023-0766

**Published:** 2023-08-09

**Authors:** Bijie Yang, Yuanyuan Liu, Feifei Xiao, Zhilong Liu, Zhe Chen, Zhigang Li, Chengfang Zhou, Mei Kuang, Yi Shu, Shan Liu, Lin Zou

**Affiliations:** Center for Clinical Molecular Medicine & Newborn Screening, Children’s Hospital of Chongqing Medical University, National Clinical Research Center for Child Health and Disorders, Ministry of Education Key Laboratory of Child Development and Disorders, Chongqing Engineering Research Center of Stem Cell Therapy, 400014, Chongqing, PR China; Chongqing Engineering Research Center of Stem Cell Therapy, Chongqing, China; Institute of Life Sciences, Chongqing Medical University, Chongqing, China; Department of Hematology, Southwest Hospital, Third Military Medical University (Army Medical University), Chongqing, China

**Keywords:** hematopoietic stem cells, self-renewal, demethylation

## Abstract

*Alkbh5* is one of the primary demethylases responsible for reversing N6-methyladenosine (m^6^A) modifications on mRNAs, and it plays a crucial role in many physiological and pathological processes. Previous studies have shown that *Alkbh5* is required for maintaining the function of leukemia stem cells but is dispensable for normal hematopoiesis. In this study, we found that *Alkbh5* deletion led to a moderate increase in the number of multiple progenitor cell populations while compromising the long-term self-renewal capacity of hematopoietic stem cells (HSCs). Here, we used RNA-seq and m^6^A-seq strategies to explore the underlying molecular mechanism. At the molecular level, *Alkbh5* may regulate hematopoiesis by reducing m^6^A modification of *Cebpa* and maintaining gene expression levels. Overall, our study unveiled an essential role for *Alkbh5* in regulating HSC homeostasis and provides a reference for future research in this area.

## 1 Introduction

In mammals, hematogenesis occurs throughout the lifetime of the animal, primarily in the bone marrow, which is driven by hematopoietic stem cells (HSCs) [1]. HSCs have extensive abilities to regenerate themselves and precipitate all cell lineages of the blood [2,3]. An essential aspect of HSCs is their capacity to remain quiescent at a steady state, which is vital for maintaining their self-renewal capacity, and their ability to initiate hematopoiesis rapidly when required [4,5]. The complex mechanisms involved in maintaining the balance between quiescence and proliferation under stress are tightly regulated [6,7], but they are not yet fully understood. Therefore, increasing knowledge of HSCs homeostasis is essential for the development of therapeutic applications involving these cells.

N6-methyladenosine (m^6^A) modifications frequently occur in mammalian messenger RNAs and are critical for various cellular processes, including hematopoiesis and leukemia development [8,9,10]. These reversible methylation events are catalyzed by the m^6^A methyltransferase complex (*Mettl3-Mettl14-Wtap*) [11] and two functionally non-redundant demethylases, α-ketoglutarate-dependent dioxygenase AlkB homolog 5 (*Alkbh5*) [12] and FTO α-ketoglutarate dependent dioxygenase (*Fto*) [13]. *Alkbh5* is a ferrous iron/2-oxoglutarate-dependent dioxygenase that can remove m^6^A methylation and is involved in mammalian RNA metabolism [12,14]. Previous work showed that *Alkbh5* is required for splicing and stabilizing long 3′-UTR mRNAs of male germ cells, which is correlated with spermatogenesis and male fertility [12,15]. There is also evidence of a role for *Alkbh5* in tumor development; *Alkbh5* regulates the self-renewal and proliferation capacity of tumor stem cells of several cancers, including glioblastoma [16], breast cancer [17], and leukemia [18,19].

Despite numerous pieces of evidence supporting *Alkbh5* in HSCs maintenance, *Alkbh5*-deficient mice showed normal hematopoiesis [18,19]. However, in our study, we found that *Alkbh5* deficiency considerably impaired normal hematogenesis under steady-state and stress conditions. Loss of *Alkbh5* led to deficiencies in sustaining HSCs quiescence, resulting in defective self-renewal capacity. Exploration of possible mechanisms underlying these observations showed that *Alkbh5* may directly regulate *Cebpa* stability by impaired m^6^A demethylation in normal hematopoiesis. Our study reveals the indispensable role of *Alkbh5* in regulating HSCs homeostasis and provides a reference for future clinical applications.

## 2 Materials and methods

### 2.1 Mice

*Alkbh5* ^*fl/fl*^ *Mx1-Cre* mice were generated by crossing *Alkbh5* ^*fl/+*^ mice with *Mx1-Cre* mice and verified by genotyping tail DNA. Mice aged 4–8 weeks were used for experiments. *In vivo* ablation of *Alkbh5* was accomplished via intraperitoneal injection of poly(I:C) (GE Healthcare, USA) at dosages of 25 µg and 10 µg/g of body weight for partial and complete ablation, respectively.

### 2.2 Flow cytometry

Single-cell suspensions from the bone marrow, thymus, and spleen were subjected to erythrocyte lysis using red blood lysis buffer (Solarbio, China) and stained with various fluorochrome- or biotin-conjugated antibodies (Table S1) before being analyzed on FACSCanto II flow cytometer (BD Biosciences, USA). For cell-cycle analysis, bone marrow cells were stained with HSCs markers and treated with the Fixation/Permeabilization Kit (BD Biosciences) according to the manufacturer. Quantitative analysis was performed using FlowJo software (V10, USA).

### 2.3 Cell isolation

Bone marrow cells were sorted by FACS AriaII flow cytometer (BD Biosciences) into distinct cell populations based on specific marker expression. The following markers were employed: Lineage negative (Lin−; Gr-1^−^Ter119^−^B220^−^CD19^−^IgM^−^IL-7R^−^CD3^−^); hematopoietic progenitor cell (HPC) (Lin^−^Sca-1^−^c-Kit^+^); murine primitive hematopoietic stem and progenitor cells (LSK) (Lin^−^Sca-1^+^c-Kit^+^); multipotent progenitor (MPP) (Lin^−^Sca-1^+^c-Kit^+^Flk2^+^CD34^+^); HSC (Lin^−^Sca-1^+^c-Kit^+^CD48^−^CD150^+^); long-term HSC (LT-HSC) (Lin^−^Sca-1^+^c-Kit^+^Flk2^−^CD34^−^); short-term HSC (ST-HSC) (Lin^−^Sca-1^+^c-Kit^+^Flk2^−^CD34^+^); common myeloid progenitor (CMP) (Lin^−^Sca-1^−^c-Kit^+^CD16/32^−^CD34^+^); granulocyte-monocyte progenitor (GMP) (Lin^−^Sca-1^−^c-Kit^+^CD16/32^+^CD34^+^); megakaryocyte-erythroid progenitor (MEP) (Lin^−^Sca-1^−^c-Kit^+^CD16/32^−^CD34^−^); and lymphoid-primed multipotential progenitor (LMPP) (Lin^−^Sca-1^+^c-Kit^+^Flk2^+^). Myeloid cells (MAC^+^), B cells (B220^+^), and T cells (CD3^+^) were sorted from the peripheral blood (PB).

### 2.4 Competitive repopulation assay

Recipient mice (CD45.1^+^) were irradiated and subsequently transplanted with bone marrow cells from donor mice (CD45.2^+^, donor types carried either *Alkbh5* ^*fl/fl*^ or *Alkbh5* ^*fl/fl*^ *Mx1-Cr*e) and competitor mice (CD45.1^+^CD45.2^+^) at a 1:1 ratio. Six weeks post-transplantation, *Alkbh5* in the recipients was knocked out by injecting poly(I:C). Six months after poly(I:C) injection, an equivalent number of bone marrow cells from the first cohort of transplanted mice were transferred into a new cohort of irradiated mice. Donor- and competitor-derived PB cells were assessed on a monthly basis after transplantation using flow cytometry.

### 2.5 5-Fluorouracil (5-FU) treatment

5-FU (Sigma-Aldrich, USA) was injected intraperitoneally at a dose of 150 mg/kg body weight once a week for 3 weeks, with daily monitoring of survival. The data were analyzed with the GraphPad Prism 6.0 software (GraphPad Software, USA).

### 2.6 Homing assay

Recipient mice underwent whole-body irradiation 24 h prior to receiving bone marrow cells (20 × 10^6^) from *Alkbh5* ^*fl/fl*^ or *Alkbh5-CKO* mice that had been stained with carboxyfluorescein diacetate succinimidyl ester (CFSE) (5 μmol/L). Six hours after cell injection, bone marrow cells were extracted from recipient mice and stained with antibodies against lineage markers and Sca-1. The percentage of CFSE^+^ cells within the Lin^−^Sca-1^+^ population was evaluated by flow cytometry.

### 2.7 RNA extraction and quantitative real-time PCR (qRT-PCR)

Bone marrow cells were sorted into different populations for total RNA extraction with RNAiso Plus (TaKaRa, China). The PrimeScript RT Reagent Kit (TaKaRa) was used for reverse transcription followed by qRT-PCR reactions with SYBR Green Master Mix (Qiagen, Germany) on a BioRad thermocycler using specific primers for *Alkbh5* and *Actin* gene: *Alkbh5* forward: 5′-CGCGGTCATCAACGACTACC-3′; *Alkbh5* reverse: 5′-ATGGGCTTGAACTGGAACTTG-3′; *Actin* forward: 5′-ACCTTCTACAATGAGCTGCG-3′; *Actin* reverse: 5′-CTGGATGGCTACGTACATGG-3′.

### 2.8 Western blot (WB)

WB analysis was performed using the standard protocols. Mouse antibody against *Alkbh5* (Sigma, USA) was used at a 1:1,000 dilution, and mouse anti-glyceraldehyde-3-phosphate dehydrogenase (GAPDH) antibody (Proteintech, USA) was used at a 1:1,000 dilution. All experiments were performed three times with GAPDH as the internal control.

### 2.9 RNA sequencing

mRNA libraries were constructed from approximately 8,000 HSCs using Smart-Seq V4 Ultra Low Input RNA Kit (TaKaRa). Fragmented DNA was barcoded with dual indexes and PCR amplified. Purification and selection of DNA fragment sizes were performed using AMPure beads. Sequencing was performed on the Illumina HiSeq 2000.

### 2.10 m^6^A sequencing

Lin^−^ cells from *Alkbh5* ^*fl/fl*^ and *Alkbh5* ^*fl/fl*^ *Mx1-Cr*e mice were sorted using FACS AriaII flow cytometer and resuspended in Trizol (RNAiso Reagent; TaKaRa). All samples were processed and sequenced at Lianchuan Biotechnology Corporation, Hangzhou, China.

### 2.11 Statistical analysis

Data processing and statistical analyses with unpaired Student’s *t*-tests were performed using GraphPad Prism v.7. Kaplan–Meier plots were analyzed with the log-rank test. P-values below 0.05 are considered statistically significant, and the levels of significance indicated as *P < 0.05, **P < 0.01, and ***P < 0.001 are compared with the control.


**Ethics statement:** All animal experiments were carried out in accordance with international ethical guidelines and the National Institutes of Health Guide concerning the Care and Use of Laboratory Animals. Animal research facilities were provided by the Third Military Medical University, China, and all animal experiments were approved by this institution (Permit Number SYXK-2017-0011).

## 3 Results

### 3.1 Generation of *Alkbh5* conditional knockout mice

**Figure 1:**
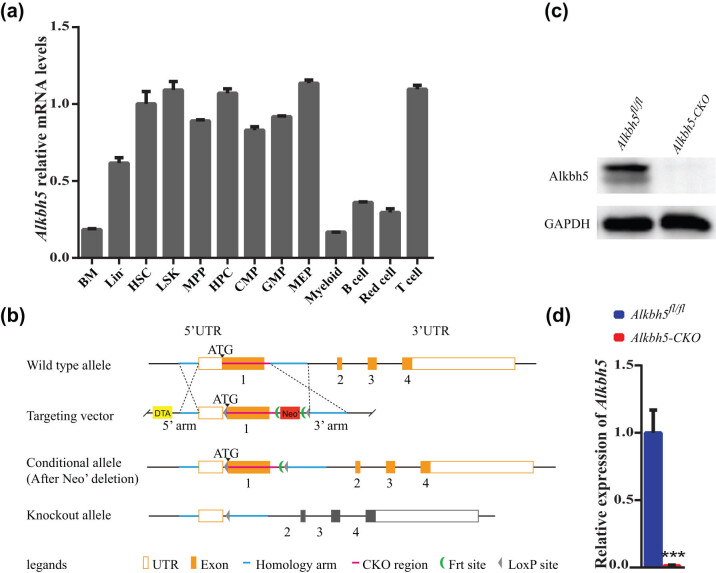
Generation of *Alkbh5* conditional knockout mice. (a) qRT-PCR analysis of *Alkbh5* in different hematopoietic cell subsets in *Alkbh5* ^*fl/fl*^ mice, including bone marrow, Lin^−^, HSCs, LSK cells, MPPs, HPCs, CMPs, GMPs, MEPs, myeloid cells, B cells, Red cells, CD4^+^ T cells, and CD8^+^ T cells. (b) Scheme for generating *Alkbh5* conditional knockout mouse. (c) Immunoblotting for *Alkbh5* and *GAPDH* in *Alkbh5* ^*fl/fl*^ and *Alkbh5* ^*fl/fl*^ *Mx1-Cr*e mice expressing indicated constructs. (d) qRT-PCR analysis showing the efficiency of *Alkbh5* deletion. *P < 0.05; **P < 0.01; ***P < 0.001.

To investigate the role of *Alkbh5* in hematopoiesis, we employed qRT-PCR to characterize *Alkbh5* expression in subgroups of primitive and mature cells within the total bone marrow. We found that *Alkbh5* was highly expressed in hematopoietic progenitor cells, including HSCs, compared to mature cells, indicating a potential role for *Alkbh5* in hematopoietic development (Figure 1a). Germline knockout of *Alkbh5* did not alter the number of hematopoietic stem and progenitor cells in 6- to 10-week-old mice [18]. Therefore, we generated a conditional knockout model (*Alkbh5* ^*fl/+*^) in which the 5′ UTR and exon 1 of *Alkbh5* are flanked with loxP sites. The *Alkbh5* ^*fl/+*^ mice were crossed with *Mx1-Cre* mice to generate control *Alkbh5* ^*fl/fl*^ mice and *Alkbh5* ^*fl/fl*^ *Mx1-Cr*e mice (Figure 1b). Cre recombinase controlled by the Mx1 promoter can be activated by synthetic double-stranded RNA (poly(I:C)) to trigger conditional deletion of the *Alkbh5* gene in *Alkbh5* ^*fl/fl*^ *Mx1-Cr*e mice (*Alkbh5-CKO*). Accordingly, *Alkbh5* deficiency in bone marrow cells was confirmed through analysis of transcript and protein levels via WB and qRT-PCR, respectively (Figure 1c and d).

### 3.2 *Alkbh5* deletion considerably accelerates hematopoietic stem and progenitor cell (HPSC) expansion in the steady state

**Figure 2:**
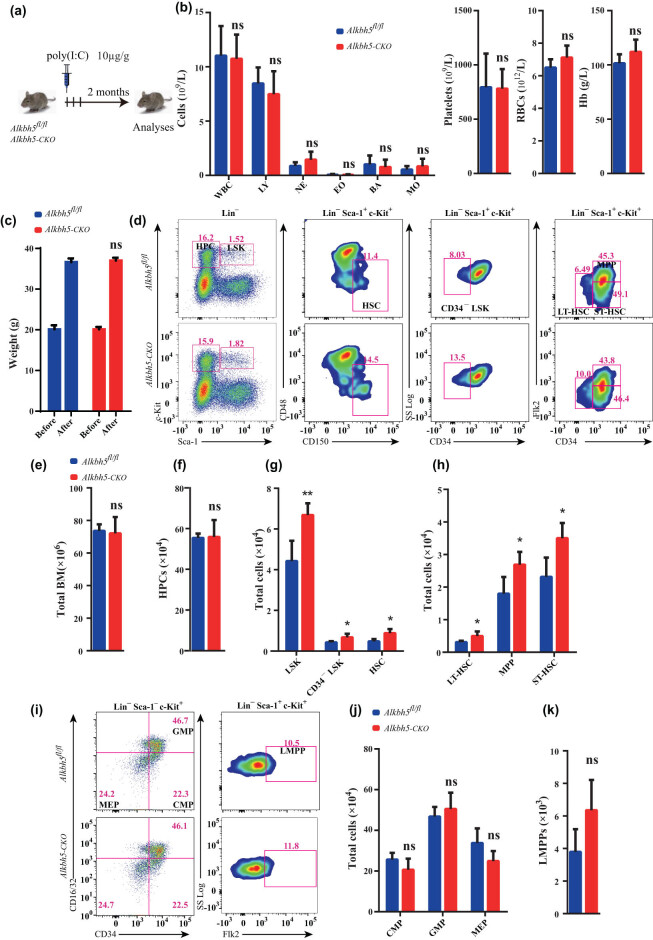
*Alkbh5* deletion considerably accelerates HSPC expansion in the steady state. (a) Experimental schematic for induction assay. (b) PB complete blood cell counts of *Alkbh5* ^*fl/fl*^ and *Alkbh5-CKO* mice at 2 months after poly(I:C) injection. WBC, white blood cells; LY, lymphocytes; NE, neutrophils; EO, eosinophils; BA, basophils; MO, monocytes; PLT, platelets; RBC, red blood cells; and Hb, hemoglobin. (c) Weight analysis of *Alkbh5* ^*fl/fl*^ and *Alkbh5-CKO* mice before and 2 months after poly(I:C) injection. (d) FACS analysis of HPCs, LSK cells, HSCs, MPPs, CD34^−^LSK cells, ST-HSCs, and LT-HSCs in bone marrow cells of *Alkbh5* ^*fl/fl*^ and *Alkbh5-CKO* mice at 2 months after poly(I:C) injection. (e) Bone marrow cells count in *Alkbh5* ^*fl/fl*^ and *Alkbh5-CKO* mice at 2 months after poly(I:C) injection. (f–h) Count of HPCs, LSK cells, HSCs, MPPs, CD34^−^LSK cells, ST-HSCs, and LT-HSCs in bone marrow cells of *Alkbh5* ^*fl/fl*^ and *Alkbh5-CKO* at 2 months after poly(I:C) injection. (i) FACS analysis of CMPs, GMPs, HSCs, MEPs, and LMPPs in bone marrow cells of *Alkbh5* ^*fl/fl*^ and *Alkbh5-CKO* mice at 2 months after poly(I:C) injection. (j and k) Counts of CMPs, GMPs, HSCs, MEPs, and LMPPs in bone marrow cells of *Alkbh5* ^*fl/fl*^ and *Alkbh5-CKO* mice at 2 months after poly(I:C) injection.

To investigate the role of *Alkbh5* in multilineage hematopoietic development, we conducted a comprehensive analysis of the PB from *Alkbh5* ^*fl/fl*^ control and *Alkbh5-CKO* mice 2 months after *Alkbh5* ablation. We observed no significant differences in differentiated cell lineages between the two groups (Figure 2a and b), nor in the body weight of the mice (Figure 2c). Furthermore, we observed no significant changes in total bone marrow cellularity or certain subpopulations (such as LMPPs and HPCs, which contain CMPs, GMPs, and MEPs) due to *Alkbh5* deletion (Figure 2d–f and i–k).

Interestingly, we found that *Alkbh5* deficiency led to a considerable increase in the total cell number of LT-HSCs, ST-HSCs, and MPPs. In addition, two separate phenotypic characterizations demonstrated that the number of CD34^−^ LSK cells and signaling lymphocyte activation molecule-hematopoietic stem cells were significantly increased in *Alkbh5-CKO* mice (Figure 2d, g and h). However, the frequency of red blood cells, B cells, T cells, and myeloid cells remained unchanged for *Alkbh5* ablation (Figure S1).

Collectively, these data indicate that *Alkbh5* may play an active role in inhibiting the expansion of multiple hematopoietic stem and progenitor cell populations.

### 3.3 Loss of *Alkbh5* decreases HSCs quiescence

**Figure 3:**
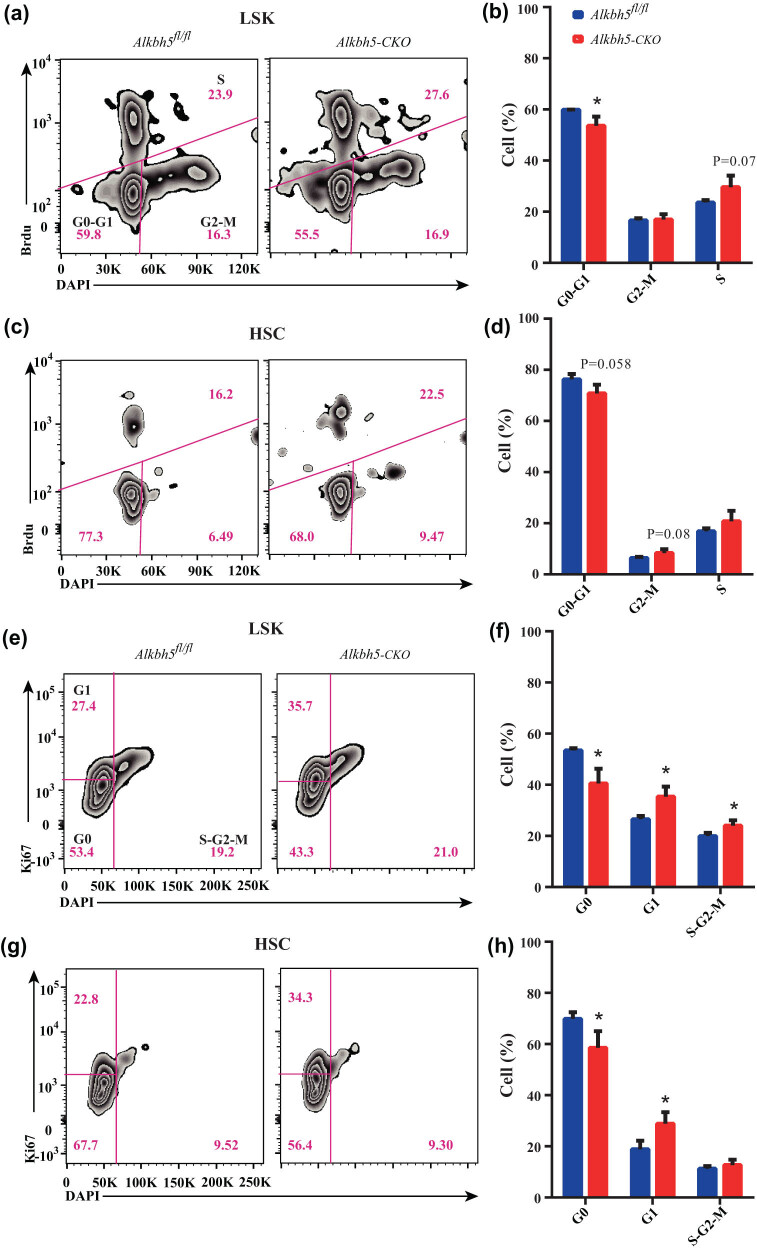
Loss of *Alkbh5* decreases HSCs quiescence. (a) FACS analysis of BrdU incorporation in LSK cells of *Alkbh5* ^*fl/fl*^ and *Alkbh5-CKO* mice. (b) Cell-cycle analysis of LSK cells in *Alkbh5* ^*fl/fl*^ and *Alkbh5-CKO* mice. (c) FACS analysis of BrdU incorporation in HSCs of *Alkbh5* ^*fl/fl*^ and *Alkbh5-CKO* mice. (d) Cell-cycle analysis of HSCs in *Alkbh5* ^*fl/fl*^ and *Alkbh5-CKO* mice. (e) FACS analysis of Ki-67 staining in LSK cells of *Alkbh5* ^*fl/fl*^ and *Alkbh5-CKO* mice. (f) Cell-cycle analysis of LSK cells in *Alkbh5* ^*fl/fl*^ and *Alkbh5-CKO* mice. (g) FACS analysis of Ki-67 staining in HSCs of *Alkbh5* ^*fl/fl*^ and *Alkbh5-CKO* mice. (h) Cell-cycle analysis of LSK cells in *Alkbh5* ^*fl/fl*^ and *Alkbh5-CKO* mice.

To determine whether *Alkbh5* controls HSCs proliferation, the cell cycle status of HSPCs from *Alkbh5* ^*fl/fl*^ and *Alkbh5-CKO* mice were analyzed. The incorporation of thymidine analog BrdU assay indicated that the percentage of BrdU^+^ HSCs was 16.9% in *Alkbh5* ^*fl/fl*^ mice and increased to 20.8% in *Alkbh5-CKO* mice, suggesting that *Alkbh5* loss promotes HSCs proliferation (Figure 3a–d). This observation is supported by Ki-67 staining, which revealed a reduced number of quiescent HSCs in *Alkbh5-CKO* mice (Figure 3e–h).

Collectively, these findings suggest that *Alkbh5 is* necessary for maintaining HSCs in a quiescence state.

### 3.4 *Alkbh5* deficiency compromises the self-renewal capacity of HSCs

**Figure 4:**
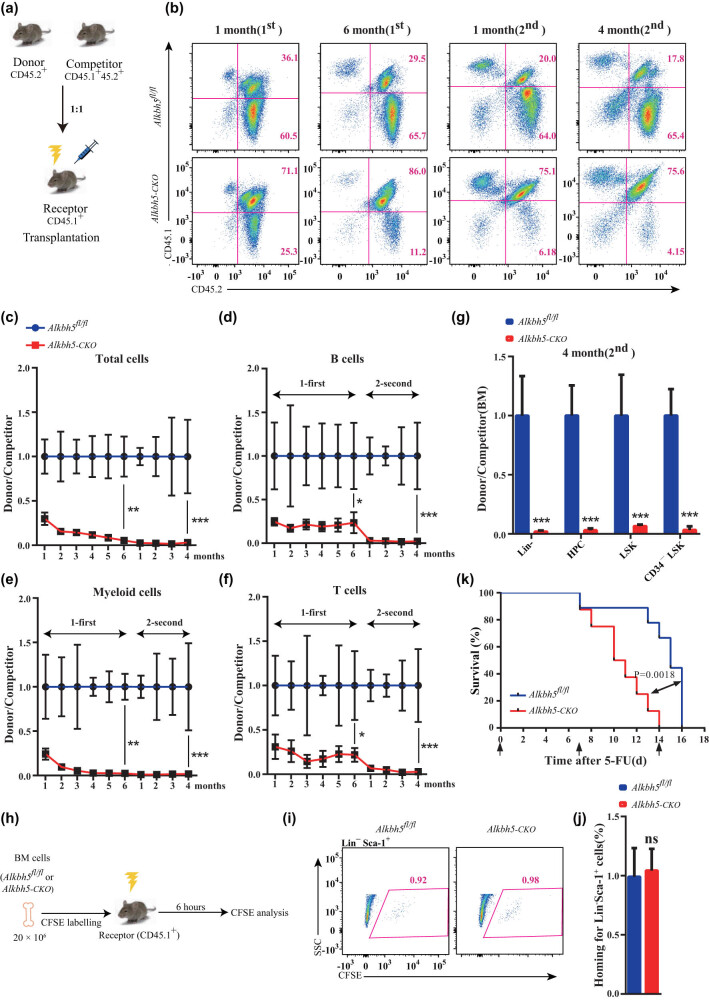
*Alkbh5* deficiency compromises the self-renewal capacity of HSCs. (a) Experimental schematic for the competitive transplantation assay. (b) FACS analysis of PB cells from recipient mice in a competitive transplantation assay. (c–f) The ratio of donor-derived CD45.2^+^ PB cells to competitor cell–derived CD45.1^+^CD45.2^+^ PB cells (donor/competitor) in the recipient mice. (g) The ratio of CD45.2^+^/CD45.1^+^CD45.2^+^ in the Lin^−^, HPCs, LSK cells, and CD34^−^LSK cells. (h) Experimental schematic for the homing assay. (i) FACS analysis of CFSE^+^ cells in bone marrow Lin^−^Sca-1^+^ cells of recipient mice. (j) The histogram indicates *in vivo* homing percentage of *Alkbh5* ^*fl/fl*^ and *Alkbh5-CKO* Lin^−^Sca-1^+^ cells in recipient mice at 6 h after transplantation. (k) Survival curve of *Alkbh5* ^*fl/fl*^ and *Alkbh5-CKO* mice following sequential 5-FU treatment.

To investigate the effect of *Alkbh5* ablation on the repopulation capacity of HSCs, we performed serial competitive transplantation assays and monitored engraftment in PB over time (Figure 4a). Our results showed that the percentage of chimeric bone marrow cells from *Alkbh5*-*CKO* mice continuously decreased, compared to their wild-type counterparts, with each transplant. This indicated a lower reconstitution capacity of *Alkbh5*-deficient HSCs (Figure 4b–f). Moreover, the percentage of chimeric myeloid, B, and T cells showed a unanimous decrease, with myeloid differentiation being more severely impaired. In addition, 4 months after the second transplant, Lin^−^ cells, HPCs, LSK cells, and CD34^−^ LSK cells derived from *Alkbh5*-deficient mice exhibited a striking reduction in their ability to repopulate the bone marrow (Figure 4g).

Despite the decreased reconstitution rate, the homing capacity of *Alkbh5*-*CKO* HSPCs was unchanged (Figure 4h–j). Furthermore, administering 5-FU treatment, which removes proliferating and mature cells, stimulated HSCs to replenish the hematopoietic system. Weekly injection of 5-FU into *Alkbh5* ^*fl/fl*^ control and *Alkbh5-CKO* mice showed that *Alkbh5*-deficient mice were unable to survive beyond the second injection, while more than 60% of *Alkbh5* ^*fl/fl*^ mice remained alive and only succumbed after the third injection (Figure 4k).

In summary, our study provides clear evidence that *Alkbh5* is required for the long-term self-renewal capacity of HSCs under stress conditions.

### 3.5 Incomplete depletion of *Alkbh5* did not impair hematopoiesis in mice

*Alkbh5* has been shown to be necessary for maintaining acute myeloid leukemia stem cell function but is expendable in normal hematogenesis [18,19]. However, our data indicate that complete ablation of *Alkbh5* affects hematopoiesis. To reconcile these differences, we attempted to replicate the work of Wong et al., where *Alkbh5* was conditionally deleted by injecting three doses of poly(I:C) (25 µg) over 6 days [19] (Figure S2a). Comparing the PB from *Alkbh5* ^*fl/fl*^ and *Alkbh5-CKO* mice, we found that the differentiated cell lineages remained unchanged (Figure S2b). Similarly, the total number of cells within the bone marrow was unaltered by analyzing *Alkbh5* ^*fl/fl*^ and *Alkbh5-CKO* mice (Figure S2d). The same is true for HSPCs populations including HPCs, LSK cells, CD34^−^ LSK cells, HSCs, LT-HSCs, ST-HSCs, MPPs, CMPs, GMPs, MEPs, and LMPPs (Figure S2c and e–j). Furthermore, the percentage of red blood cells, B cells, T cells, and myeloid cells is unaltered between the two groups of mice (Figure S3). However, WB data indicated an incomplete knockout of *Alkbh5* in the *Alkbh5-CKO* mice (Figure S2k).

Therefore, our data indicate that incomplete deletion of *Alkbh5* has no apparent effect on multilineage hematopoiesis.

### 3.6 *Alkbh5* deficiency inhibits *Cebpa* signaling

**Figure 5:**
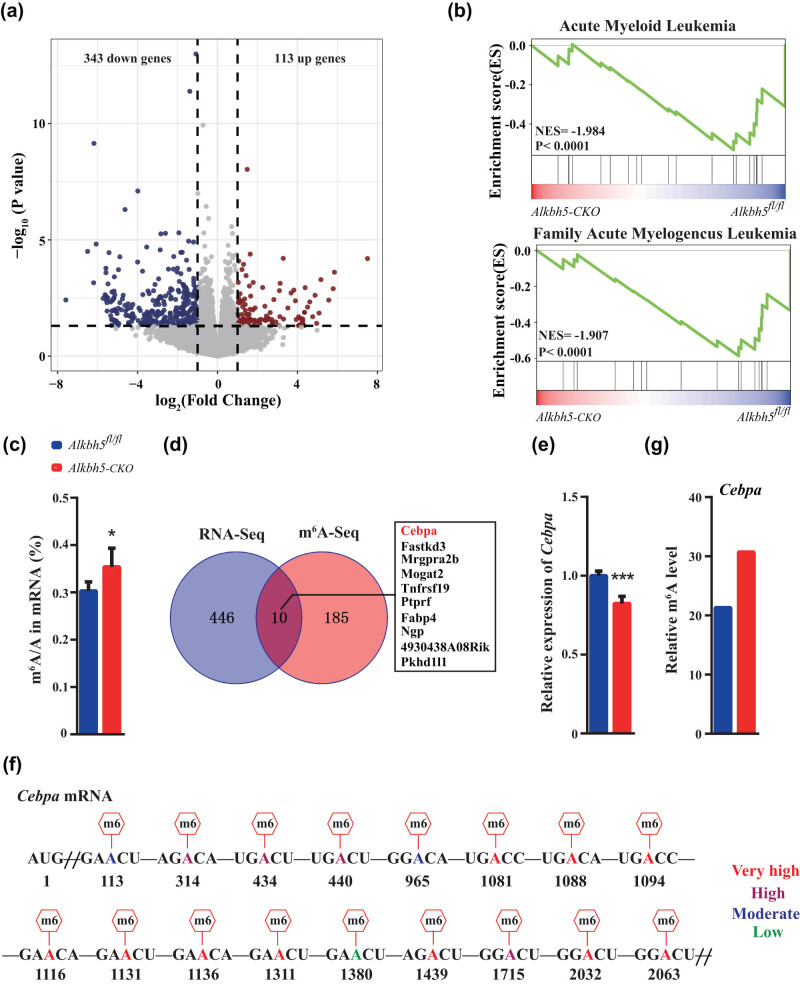
*Alkbh5* deficiency inhibits *Cebpa* signaling. (a) Representative scatters of upregulated genes or downregulated genes by 1.5-fold or more in *Alkbh5-CKO* HSCs compared with *Alkbh5* ^*fl/fl*^ HSCs. (b) GSEA of the selected gene sets. (c) m^6^A-seq shows the total m^6^A modification in Lin^−^ cells of *Alkbh5* ^*fl/fl*^ and *Alkbh5-CKO* mice. (d) Integrative analysis of RNA-seq and m^6^A-seq to identify transcriptome-wide potential targets of *Alkbh5* in HSCs. (e) RNA-seq showed the expression level of *Cebpa* transcript in HSCs of *Alkbh5* ^*fl/fl*^ and *Alkbh5-CKO* mice. (f) 17 Potential m^6^A sites of *Cebpa* mRNA predicted by SRAMP program. (g) m^6^A-seq shows changes in *Cebpa* transcript m^6^A modification in Lin^−^ cells from *Alkbh5* ^*fl/fl*^ and *Alkbh5-CKO* mice.

To uncover the mechanism by which *Alkbh5* regulates HSCs homeostasis and function, we performed RNA sequencing (RNA-seq) on freshly sorted HSCs and found that 113 were upregulated and 343 genes were downregulated in *Alkbh5-CKO* HSCs compared to *Alkbh5* ^*fl/fl*^ HSCs (fold change >1.5 and *P* value <0.05; Figure 5a). Gene-set enrichment analysis (GSEA) indicated that the downregulated genes were enriched for acute myeloid leukemia and family acute myelogenous leukemia genes (Figure 5b). As *Alkbh5* is a major m^6^A demethylase, we hypothesized that *Alkbh5* may regulate HSCs homeostasis by demethylating target genes. To identify these targets, we sorted Lin^−^ cells and performed m^6^A sequencing (m^6^A-seq) and found that m^6^A enrichment of total mRNA is significantly increased in *Alkbh5-CKO* mice (Figure 5c). Furthermore, we observed significant changes in 51 hypo-down genes, 136 hypo-up genes, 13 hyper-down genes, and 42 hyper-up genes in *Alkbh5-CKO* cells (fold change >1.2 and *q* value <0.05; Table S2) and identified 10 potential target genes through integrative analysis of RNA-seq and m^6^A-seq (Figure 5d). Of these, we focused on *Cebpa,* as its m^6^A level significantly upregulated, while its RNA expression decreased in *Alkbh5-CKO*. Previous reports showed that *Cebpa* maintains the HSCs self-renewal and inhibits progenitor population expansion [20]. We detected a significant decrease in the expression of *Cebpa* in *Alkbh5-CKO* HSCs, indicating that *Alkbh5* directly regulates *Cebpa* (Figure 5e), and identified 17 potential m^6^A modification sites on *Cebpa* using the sequence-based SRAMP m^6^A modification site predictor (http://www.cuilab.cn/sramp) (Figure 5f and Figure S4). We also found compared to *Alkbh5* ^*fl/fl*^ Lin^−^ cells, *Cebpa* mRNA from *Alkbh5-CKO* Lin^−^ cells have increased m^6^A modifications (Figure 5g), and m^6^A-seq showed that the m^6^A modification on 5′ UTR of *Cebpa*. Our findings suggest that *Alkbh5* deficiency leads to an increased m^6^A enrichment and reduced RNA expression of *Cebpa*, ultimately leading to the activation of the programs promoting HSCs proliferation and impaired self-renewal capacity.

## 4 Discussion

Previous evidence has suggested that *Alkbh5* plays a crucial role in leukemogenesis but is dispensable for hematopoiesis [18,19]. In this study, we provided alternative evidence that challenges these assumptions and supports the notion that *Alkbh5* sustains HSCs self-renewal and inhibits the expansion of multiple HSPC populations. However, incomplete ablation of *Alkbh5* had minimal effect on normal hematopoiesis, suggesting that even a very low level of *Alkbh5* expression is sufficient for maintaining HSCs quiescence. Mechanistically, *Alkbh5*, as a significant m^6^A demethylase, may regulate these homeostatic functions in the hematopoietic system by decreasing the m^6^A modification of *Cebpa*, thereby maintaining the expression of this gene.

Differences in experimental models may account for the discrepancy between our results and previous works [18,19] regarding the role of *Alkbh5* in normal hematopoiesis. Shen et al. generated germline knockout of *Alkbh5* using CRISPR-Cas9, resulting in a whole-body deletion of 19 amino acids from the protein [18]. In contrast, we specifically knocked out *Alkbh5* in the hematopoietic system of adult mice using a Cre-*lox*P system. Germline deletion of the protein may permit compensatory mechanisms to mask the role of *Alkbh5* in hematopoiesis. Wang et al. used a similar conditional knockout system to ours, wherein poly(I:C) was used to stimulate the excision of exon 1 of *Alkbh5* [19]. Although they validated the knockout efficiency of *Alkbh5* by qRT-PCR, the protein level was not verified [19]. In our model, poly(I:C)-induced exon 1 knockout of *Alkbh5* causes a frame-shift mutation that completely deletes *Alkbh5* at the protein level. When we used the same poly(I:C) dose reported by Wang et al., the knockdown of Alkbh5 protein was incomplete, and the phenotype of these mice was consistent with the results of Wang et al. These findings suggest that the timing and efficiency of *Alkbh5* knockout are important in determining the effect of this gene on hematopoiesis and highlight the need for further research in this area.

In our study, we have identified 17 potential m^6^A modification sites on *Cebpa*. According to Su et al., an increase in m^6^A modification on *Cebpa* mRNA can be detected by *Ythdf2*, which leads to a decrease in the stability and expression level of *Cebpa* [21]. The m^6^A reader is responsible for the fate of RNA, with *Ythdf2* mainly promoting the degradation of m^6^A-tagged mRNAs [22]. Given the presence of m^6^A modification on the 5′ UTR of *Cebpa*, we hypothesize that increased m^6^A modification at site 5′ UTR of *Cebpa* mRNA may be recognized by *Ythdf2*, leading to a reduction in stability and expression of *Cebpa*. However, further experiments are required to validate this hypothesis.

Wang et al. and Shen et al. have identified two signaling pathways involving *Alkbh5*/m^6^A/TACC3 and the *Kdm4c*/*Alkbh5* in acute myeloid leukemia development [18,19]. We have shown that *Alkbh5* may play an important role in regulating HSCs self-renewal and the maintenance of HSPC populations by acting on *Cebpa* as a potential target. There are likely to be many other signaling pathways through which *Alkbh5* mediates its functions.

m^6^A modification is a dynamic process facilitated by m^6^A-binding or “reader” proteins, such as *Ythdf1/2/3* and *Igf2bp1/2/3*, which can lead to the regulation of mRNA stability [23,24]. Inactivation of *Ythdf2* has been shown to increase HSCs expansion [25,26], while a lack of *Ythdf2* could reduce the population of quiescent HSCs [27]. *Alkbh5* may also regulate normal hematopoiesis through these signaling pathways, although further research is needed to confirm this.

In summary, our finding provides evidence that *Alkbh5* plays a crucial role in maintaining the inherent self-renewal capacity of HSCs and prevents the expansion of multiple hematopoietic stem and progenitor cell populations. *Alkbh5* may regulate normal hematopoiesis directly by maintaining the expression of *Cebpa* through the reduction of m^6^A modification.

## Supplementary Material

Supplementary Figure

Supplementary Table 1

Supplementary Table 2
