# Supplementary material for: Alkbh5 plays indispensable roles in maintaining self-renewal of hematopoietic stem cells
Source: Open Med (Wars). 2023 Aug 9;18(1):20230766. doi: 10.1515/med-2023-0766 (PMC10426271; doi:10.1515/med-2023-0766)
Supplement: Supplementary Figure [file med-2023-0766-sm1.pdf]

Supplementary material

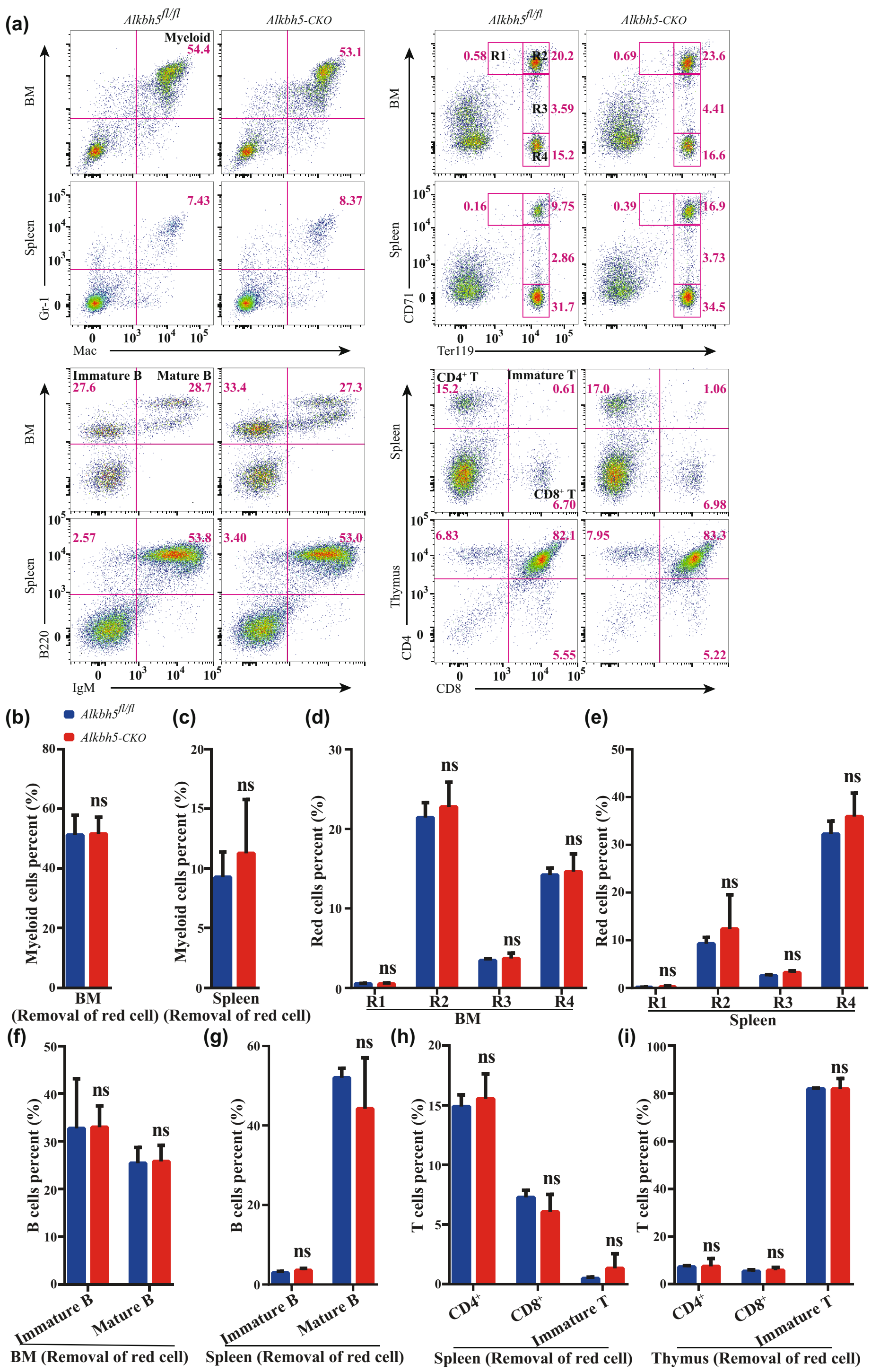

Figure S1: *Alkbh5* deletion impairs hematopoiesis in mice. (a) FACS analysis of myeloid cells (Mac^+Gr1^+), red cells (R1:$Ter119^{med}CD71^{high}$, R2:$Ter119^{high}CD71^{high}$, R3: $Ter119^{high}CD71^{med}$, R4: $Ter119^{high}CD71^{low}$), B cells (immature B: IgM^-B220^+; mature B: IgM^+B220^+), and T cells (immature T:$CD8a^+CD4^+$; $CD4^+$T; $CD8^+$T) in bone marrow, spleen, and thymus of *Alkbh5*$^{fl/fl}$ and *Alkbh5-CKO* mice at 2 months after poly(I:C) injection. (b and c) Percent analysis of myeloid cells in bone marrow and spleen cells. (d and e) Percent analysis of red cells in bone marrow and spleen cells. (f and g) Percent analysis of B cells in bone marrow and spleen cells. (h and i) Percent analysis of T cells in spleen and thymus cells.

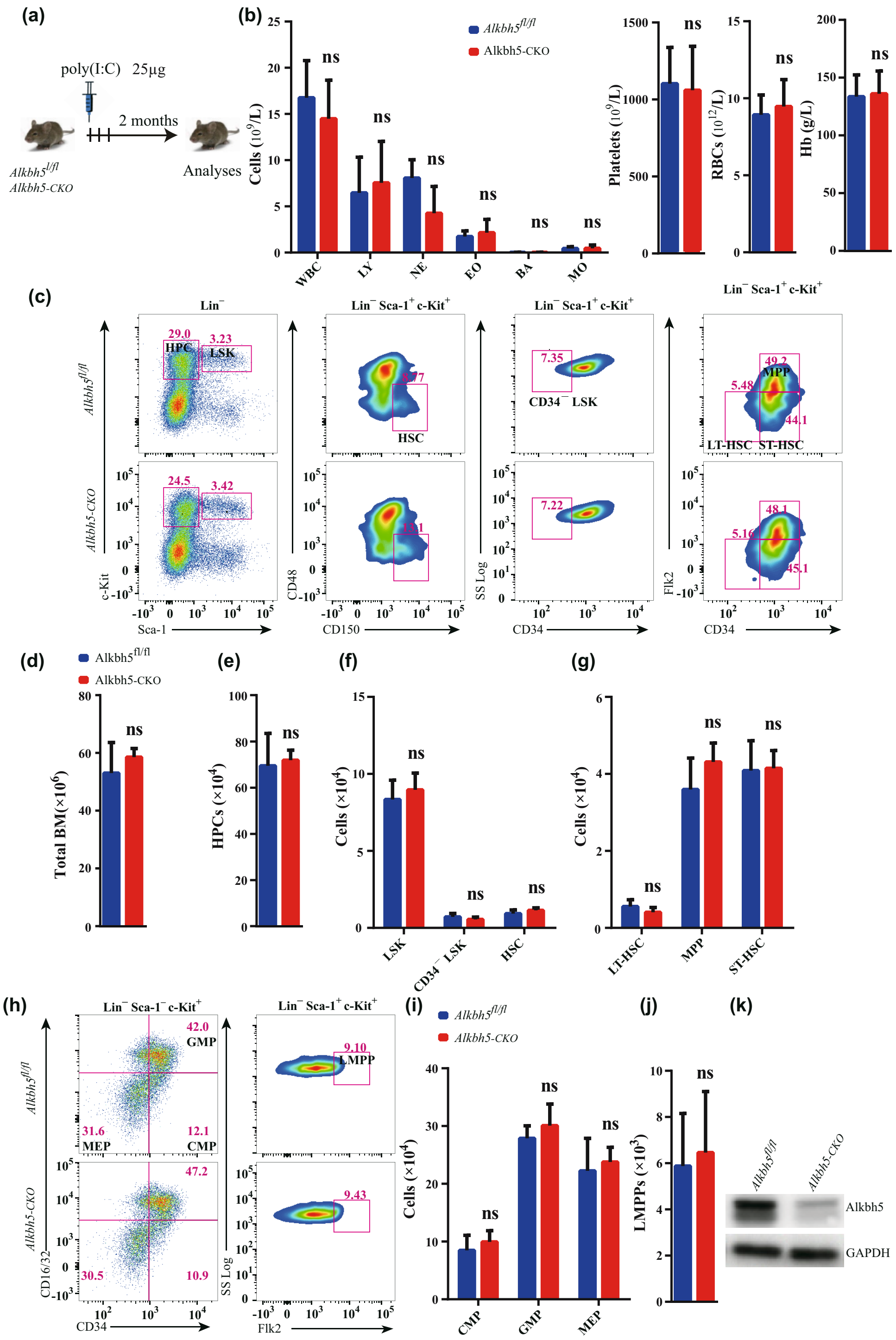

Figure S2: Incomplete depletion of *Alkbh5* did not impair hematopoiesis in mice. (a) Experimental schematic for induction assay. (b) PB complete blood cell counts of *Alkbh5*$^{fl/fl}$ and *Alkbh5-CKO* mice at 2 months after poly(I:C) injection. (c) FACS analysis of HPCs, LSK cells, HSCs, MPPs, CD34^{-}LSK cells, ST-HSCs, and LT-HSCs in bone marrow cells of *Alkbh5*$^{fl/fl}$ and *Alkbh5-CKO* mice at 2 months after poly(I:C) injection. (d) Bone marrow cells count in *Alkbh5*$^{fl/fl}$ and *Alkbh5-CKO* mice at 2 months after poly(I:C) injection. (e-g) Count of HPCs, LSK cells, HSCs, MPPs, CD34^{-}LSK cells, ST-HSCs, and LT-HSCs in bone marrow cells of *Alkbh5*$^{fl/fl}$ and *Alkbh5-CKO* mice at 2 months after poly(I:C) injection. (h) FACS analysis of CMPs, GMPs, HSCs, MEPs and LMPPs in bone marrow cells of *Alkbh5*$^{fl/fl}$ and *Alkbh5-CKO* mice at 2 months after poly(I:C) injection. (i and j) Count of CMPs, GMPs, HSCs, MEPs, LMPPs and CLPs in bone marrow cells of *Alkbh5*$^{fl/fl}$ and *Alkbh5-CKO* mice at 2 months after poly(I:C) injection. (k) Immunoblotting for *Alkbh5* and *GAPDH* in *Alkbh5*$^{fl/fl}$ and *Alkbh5-CKO* mice expressing indicated constructs.

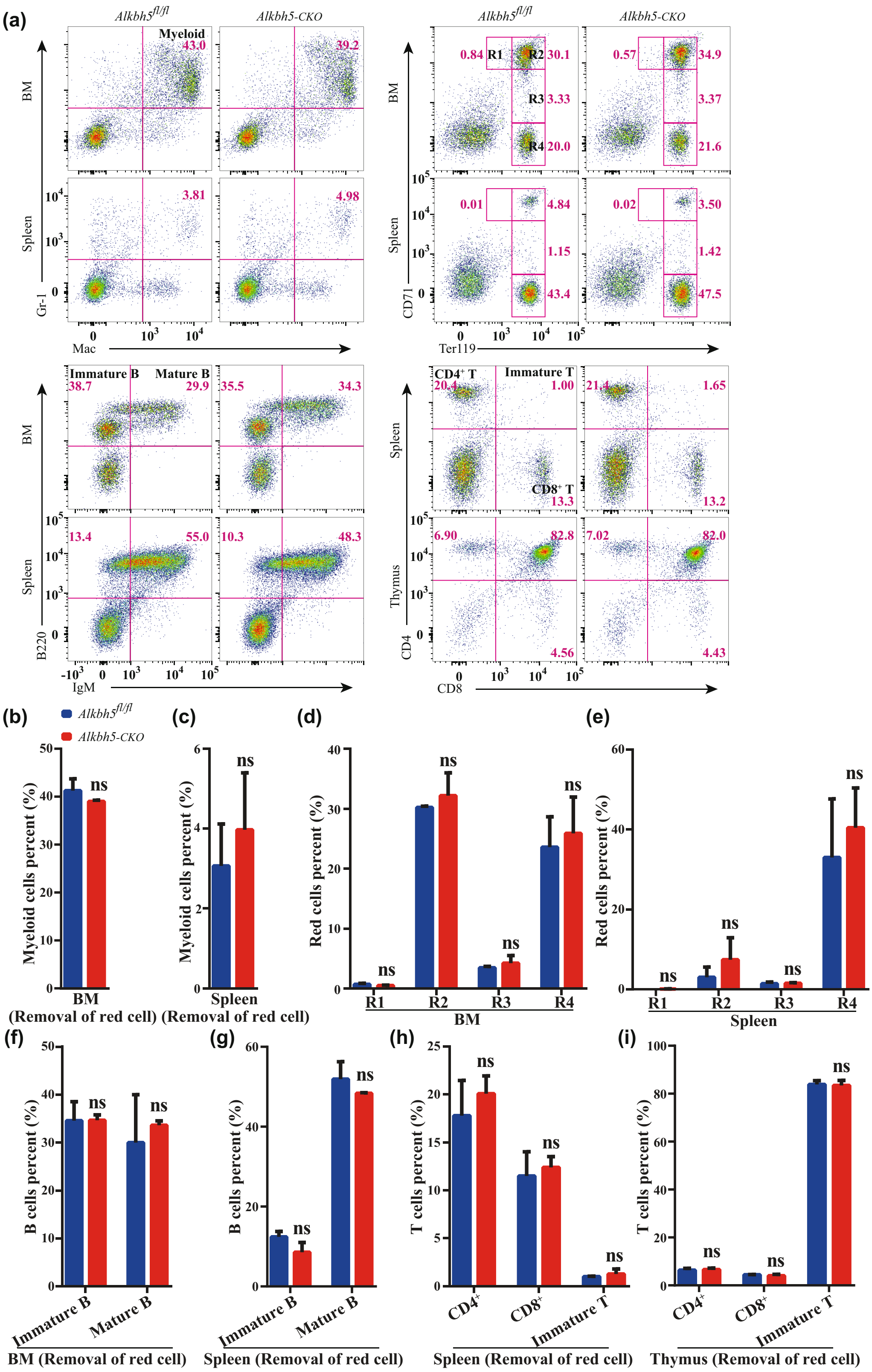

Figure S3: Incomplete deletion of *Alkbh5* did not affect the hematopoietic of mice. (a) FACS analysis of myeloid cells, red cells, B cells and T cells in bone marrow, spleen, and thymus of *Alkbh5*$^{fl/fl}$ and *Alkbh5-CKO* mice at 2 months after poly(I:C) injection. (b and c) Percent analysis of myeloid cells in bone marrow and spleen cells. (d and e) Percent analysis of red cells in bone marrow and spleen cells. (f and g) Percent analysis of B cells in bone marrow and spleen cells. (h and i) Percent analysis of T cells in spleen and thymus cells.

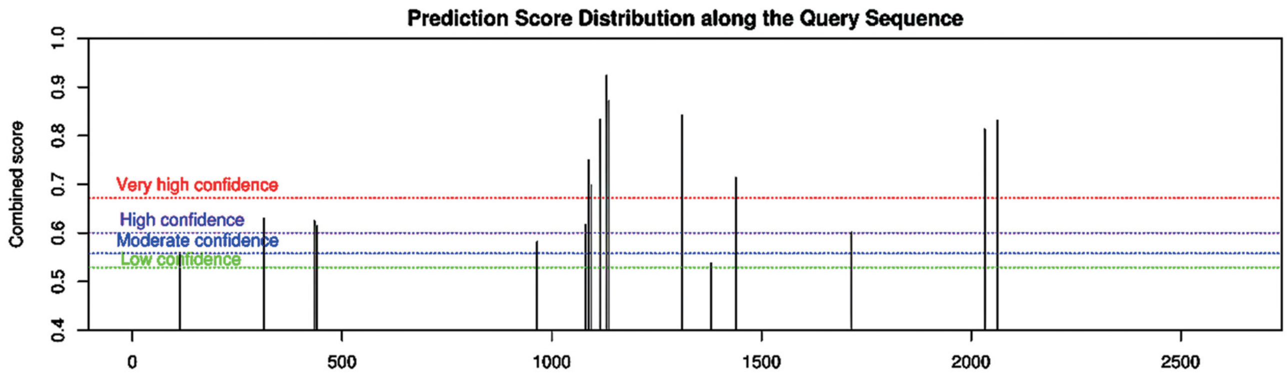

Figure S4: The potential m^{6}A modification sites of *Cebpa* mRNA by SRAMP program.
